# Cost-Related Medication Nonadherence (CRN) on Healthcare Utilization and Patient-Reported Outcomes: Considerations in Managing Medicare Beneficiaries on Antidepressants

**DOI:** 10.3389/fphar.2021.764697

**Published:** 2021-12-07

**Authors:** Abdulrahman A. Alnijadi, Jing Yuan, Jun Wu, Minghui Li, Z. Kevin Lu

**Affiliations:** ^1^ Department of Clinical Pharmacy and Outcomes Sciences, University of South Carolina, Columbia, SC, United States; ^2^ Department of Pharmacy Practice, College of Clinical Pharmacy, King Faisal University, Al-Ahsa, Saudi Arabia; ^3^ Department of Clinical Pharmacy and Pharmacy Practice, School of Pharmacy, Fudan University, Shanghai, China; ^4^ Department of Pharmaceutical and Administrative Sciences, Presbyterian College, Clinton, SC, United States; ^5^ Department of Clinical Pharmacy and Translational Science, University of Tennessee Health Science Center, Memphis, TN, United States

**Keywords:** cost-related medication nonadherence, healthcare utilization, patient reported outcomes, older adults, medicare beneficiaries, antidepressants

## Abstract

**Background:** Many patients face a financial burden due to their medications, which may lead to poor health outcomes. The behaviors of non-adherence due to financial difficulties, known as cost-related medication non-adherence (CRN), include taking smaller doses of drugs, skipping doses to make prescriptions last longer, or delaying prescriptions. To date, the prevalence of CRN remains unknown, and there are few studies about the association of CRN on self-reported healthcare utilization (Emergency room (ER) visits and outpatient visits) and self-reported health outcomes (health status and disability status) among older adults taking antidepressants.

**Objectives:** The objectives were to 1) examine the CRN prevalence, and 2) determine the association of CRN on self-reported healthcare utilization and self-reported health outcomes.

**Methods:** This study was a cross-sectional study of a sample of older adults from the Medicare Current Beneficiary Survey (MCBS) who reported having used antidepressants in 2017. Four logistic regressions were implemented to evaluate the association of CRN, and self-reported healthcare utilization and self-reported health outcomes.

**Results:** The study identified 602 participants who were Medicare beneficiaries on antidepressants. The prevalence of CRN among antidepressant users was (16.61%). After controlling for covariates, CRN was associated with poorer self-reported outcomes but not statistically significant: general health status [odds ratio (OR): 0.67; 95% confidence interval (CI): 0.39–1.16] and disability status (OR: 1.34; 95% CI: 0.83–2.14). In addition, CRN was associated with increased outpatient visits (OR: 1.89; 95% CI: 1.19–3.02), but not associated with ER visits (OR: 1.10; 95% CI: 0.69–1.76).

**Conclusion:** For Medicare beneficiaries on antidepressants, CRN prevalence was high and contributed to more outpatient visits. The healthcare provider needs to define the reasoning for CRN and provide solutions to reduce the financial burden on the affected patient. Also, health care providers need to consider the factors that may enhance patient health status and healthcare efficiency.

## 1 Background

Antidepressant drugs aim to relieve the symptoms of depression and are also used for many conditions of mental well-being and long-term pain management (NHS Choices, 2021). Antidepressants act by resolving neurotransmitter’s chemical imbalance in the brain (Andrade and Rao, 2010). The chemical imbalances in the brain contribute to changes in the patient’s behavior and mood (Harvard Health, 2019). Examples of these neurotransmitters are serotonin, dopamine and noradrenaline, and norepinephrine (Khushboo and Sharma, 2017). There are many antidepressant drug classes available to help with the chemical imbalance in the brain. The most commonly prescribed classes are Selective Serotonin Reuptake Inhibitors (SSRIs), Serotonin and Noradrenaline Reuptake Inhibitors (SNRIs), Tricyclic Antidepressants (TCAs), Monoamine Oxidase Inhibitors (MAOIs), and Atypical Antidepressants (Antidepressants, 2021; Ogbru, 2021). Approximately 20% of older adults use antidepressants in a month, according to the National Center for Health Statistics (NCHS) (Products Data Briefs Number, 2020)*.*

Older adults are prone to financial burden and increased health care expenditures linked to polypharmacy and comorbidities (Nobili et al., 2011). Psychiatric drugs are among the most expensive drugs (Bartels, 2003). Medications are priced differently based on whether they are generic, brand-name, or mail-order, and they are divided into five tiers (Behring, 2020). When a drug is placed in a higher tier, the patient will pay a higher copayment or coinsurance (Medicare, 2021). The difference in prices will be applicable for all medications, including antidepressant prescriptions. Many patients face a serious financial burden due to medications, contributing to medication non-adherence to save money. In addition, there is significant data on how Medicare Part D and the coverage gap impact utilization and out-of-pocket expenditures (Park and Martin, 2016).

Antidepressants are the third among prescription drugs and the fourth most commonly sold drug in the United States, with up to 10% of adults taking at least one prescription (Pratt et al., 2011; Davey and Chanen, 2016). It is critical to determine the extent of non-adherence with these drugs. Non-adherence happens when patients skip 20% or higher of the antidepressant drug (ten Doesschate et al., 2009). It is believed that comorbid conditions, patient characteristics, patient behaviors, and patient education can be significant factors that may influence medication adherence (Pampallona et al., 2002; Prukkanone et al., 2010). Non-adherence with medication may pose a higher risk to older adults, resulting in poorer outcomes compared to younger populations (Hughes, 2004; Banning, 2008). For antidepressant drugs, there is a clear association between non-adherence and deterioration of patient clinical and economic outcomes (Ho et al., 2016). In addition, medication non-adherence may lead to health complications that result in an economic strain on the health care system, and it is more problematic, particularly for patients with chronic disorders such as psychiatric illnesses like depression (Dunbar-Jacob and Mortimer-Stephens, 2001; Weiden et al., 2004).

Non-adherence behavior caused by financial challenges is referred to as cost-related medication non-adherence (CRN) (Lee et al., 2018). The Medicare Part D introduced in 2006 was fairly successful in lowering CRN levels. Nevertheless, CRN was not completely solved, and CRN was reported among Medicare beneficiaries in subsequent years (Madden, 2008; Kennedy et al., 2011). Cost is the major cause of non-adherence for those taking antidepressants (Piette et al., 2004). Even though numerous policies (for example, Part D) have been implemented to reduce CRN, the CRN of the older adults using antidepressants remains unclear. Also, we have limited knowledge of how CRN influences individuals with varying socioeconomic factors. Acknowledging the contributing factors associated with CRN among Medicare beneficiaries on antidepressants will assist stakeholders and health care providers in order to assess CRN rates in older adults. The assessment will allow for better CRN management to overcome this issue. In addition, consider the factors that may enhance patient health status and healthcare efficiency. Therefore, the objectives of this study were to 1) examine the CRN prevalence, and 2) determine the association of CRN on healthcare utilization and self-reported health outcomes.

## 2 Methods

### 2.1 Data Source

We used the data from the Medicare Current Beneficiaries Survey (MCBS) in 2017. The MCBS is a longitudinal rotating panel survey funded by the Centers for Medicare and Medicaid Services (CMS). Medicare beneficiaries were surveyed for twelve rounds, three data collection periods per year, and followed up to 4 years. The MCBS data is ideal for this analysis, as it covers almost all applications in healthcare for eligible patients in all areas of healthcare services. In particular, the MCBS provides information on health status, access to healthcare, insurance coverage, out-of-pocket expenses, financial resources, and socio-economic and demographic characteristics of the entire beneficiary of Medicare (MCBS Methodology Report, 2016).

### 2.2 Study Population

The survey respondents were included in the study if they were 65 years or older and used antidepressants based on Medicare Part D claims. Participants were excluded from the study if they were eligible for Medicare due to End-Stage Renal Disease (ESRD) or if they were enrolled in Health Maintenance Organization (HMO) plans.

The cost of healthcare for individuals with impairments such as ESRD is more than twice as expensive as those with temporary or no disabilities (Pumkam et al., 2013). Individuals with ESRD will be sicker than included participants, which may result in a higher financial burden and more hospital visits. Excluding these people prevents extreme or outlier observations from influencing the regression result. HMO members had no data from claims on various health problems in the MCBS survey data (Raghunathan et al., 2020). Thus, they must be excluded from the study.

### 2.3 Measurements

#### 2.3.1 Dependent Variables

Self-reported health outcomes were related to the quality-of-life metrics reported in the MCBS, including health status (Excellent, Very good, Good, Fair and Poor) and disability status (No disability, One disability, Two or more disabilities) (Medicare Current Beneficiary Survey, 2017). We used binary outcomes for health status (Great health status and Poor health status) and disability status (No disability and one or more disability). In MCBS, we have included four related disability indicators: any difficulties with concentration/remembering/deciding, walking/climbing stairs, dressing/bathing, and difficulties in doing errands (Medicare Current Beneficiary Survey, 2017). Self-reported healthcare utilizations were defined as ER and hospital outpatient visits (Yes or No), as reported through the MCBS survey.

#### 2.3.2 Independent Variables

CRN was calculated based on Yes or No responses to any of these four survey questions: taking smaller doses of drugs, skipping doses to make medications last longer, delaying prescriptions because of cost, and not getting prescriptions because they cost too much (Pierre-Jacques et al., 2008; Zivin et al., 2009). Survey participants’ characteristics, including gender, age, race/ethnicity, marital status, census region, residence, income, and education. We also calculated the Charlson comorbidity index (CCI) for the number of comorbidities.

### 2.4 Statistical Analysis

We conducted four models of logistic regression, two models for the association of CRN on self-reported healthcare utilization and two models for the association of CRN on self-reported health outcomes. For all four models, the covariates were the same (demographics, socioeconomic status, and health status). The key independent variable was the same for both objectives: CRN. The key dependent variables were different depending on the objectives, including whether health outcomes (health status and disability status) or patient healthcare utilization (ER visits and outpatient visits) were used.

All the data analyses were carried out by using version 9.4 of Statistical Analysis Systems (SAS) Software.

## 3 Results

**FIGURE 1:**
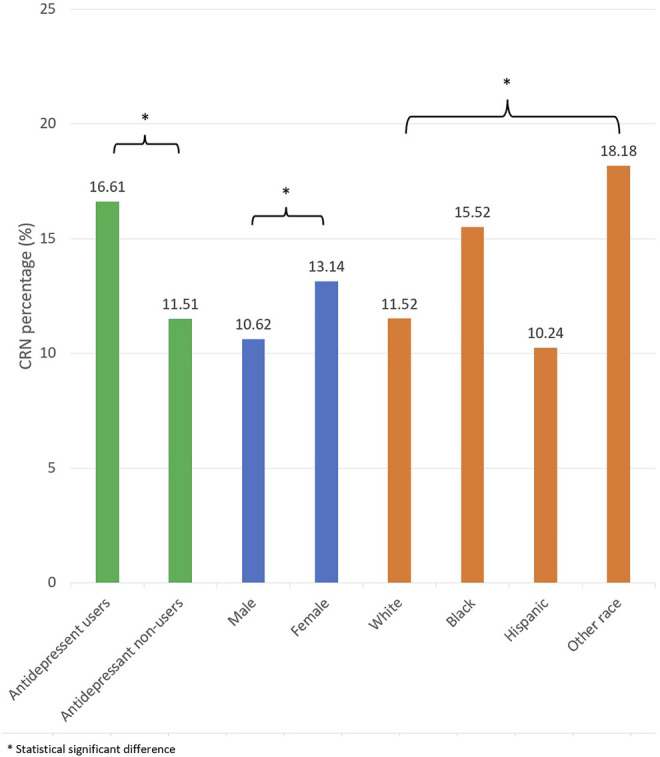
CRN prevalence by antidepressant use for racial/ethnicity and gender.

The study identified 602 participants who were Medicare beneficiaries on antidepressants. For the CRN prevalence by antidepressant use, there were 16.61% of participants reported CRN. Females reported CRN more than males (13.14 vs. 10.62%). For the prevalence by racial/ethnicity, 15.52% were African American, 11.52% were White, 10.24% were Hispanic, and 18.18% other races (Figure 1).

Table 1 described the characteristics of the study sample. For the self-reported health outcomes, 78.24% of participants reported superior general health status, and 50.66% reported no disability. For patient healthcare utilization, 43.19% of participants visited outpatient and 41.53% visited ER. For the demographic characteristic, 45.18% of the participants were age between 75 and 84 years old; 73.42% were female; 89.04% were White; 6.48% were African American; 2.99% were Hispanic; 41.69% were single; 40.70% of participants lived in the South; 74.92% were in metropolitan residence. Regarding socioeconomic status, 66.95% were income higher than $20,000 US dollars; 44.35% attended some college or possessed a degree. For the health status characteristics, 43.52% of participants have reported one or more comorbidities. For the antidepressant classes, 58.31% were SSRI users, 14.45% were SNRI users, 5.98% were TCAs users, and 31.26% used other antidepressants.

**TABLE 1:** Baseline characteristic of the study sample.

Characteristics	Antidepressant users (*n* = 602)	
	*n*	%
**CRN**		
Yes	100	16.61
No	502	83.39
**General health status**		
Great health status	471	78.24
Poor health status	131	21.76
**Disability status**		
No	305	50.66
one or more	297	49.34
**Outpatient visit**		
Yes	260	43.19
No	342	56.81
**ER visit**		
Yes	250	41.53
No	352	58.47
**Age group**		
65–74	222	36.88
75–84	272	45.18
85+	108	17.94
**Gender**		
Female	442	73.42
Male	160	26.58
**Race/ethnicity**		
Non-Hispanic White	536	89.04
Non-Hispanic Black	39	6.48
Hispanic	18	2.99
Others	—	—
**Education**		
Less than high school	199	33.06
High school graduate	136	22.59
Some college degree	93	15.45
College graduate	174	28.90
**Marital status**		
Single	251	41.69
Married	124	21.10
Widowed	224	37.21
**Family income per year (US$)**		
<10,000 per year	54	8.97
10,001–20,000 per year	145	24.09
20,001–40,000 per year	173	28.74
≥40,001 per year	230	38.21
**Census region**		
Northeast	102	16.94
Midwest	148	24.58
South	245	40.70
West	107	17.77
**Residence**		
Non-metropolitan	151	25.08
Metropolitan	451	74.92
**CCI**		
0	89	14.78
1	61	10.13
2+	201	33.39
Unknown	251	41.69
**Antidepressant class**		
SSRIs	351	58.31
SNRIs	87	14.45
TCAs	36	5.98
MAOIs	—	—
Other classes	128	21.26

CRN, cost-related medication nonadherence; CC, charlson comorbidity index; ER, emergency room; SSRIs, selective serotonin reuptake Inhibitors; SNRIs, serotonin and noradrenaline reuptake inhibitors; TCAs, tricyclic antidepressants; MAOIs, monoamine oxidase inhibitors.

The factors associated with general health status, after adjusting for possible confounders, we discovered that Medicare beneficiaries with antidepressants who had some college degree [odds ratio (OR): 2.48; 95% confidence interval (CI): 1.23–5.02], had a graduate degree (OR: 3.01; 95% CI: 1.57–5.78), had an income of $10,001-$20,000 per year (OR: 1.09; 95% CI: 0.52–2.28), had an income of $20,001-$40,000 per year (OR: 1.90; 95% CI: 0.85–4.25), and had income more than $40,000 per year (OR: 3.01; 95% CI: 1.22–7.42), and were in the South region (OR: 2.01; 95% CI: 1.08–3.75) were more likely to report a superior general health status (Table 2). On the contrary, among antidepressant users who were male (OR: 0.50; 95% CI: 0.30–0.83), were Africans Americans (OR: 0.46; 95% CI: 0.22–0.97), had less than a high school of education (OR: 0.86; 95% CI: 0.49–1.50), had one comorbidity (OR: 0.34; 95% CI: 0.13–0.87), and had two or more comorbidities (OR: 0.32; 95% CI: 0.14–0.72) were more likely to report an inferior general health status. Furthermore, the factors associated with disability status, antidepressant users who had some college degree (OR: 0.60; 95% CI: 0.35–1.02), had a graduate degree (OR: 0.61; 95% CI: 0.38–0.97), had an income of $10,001-$20,000 per year (OR: 0.93; 95% CI: 0.44–1.96), had an income of $20,001-$40,000 per year (OR: 0.50; 95% CI: 0.23–1.06), had an income of $40,000 or more (OR: 0.40; 95% CI: 0.18–0.89) were less likely to report a disability (Table 2). On the other hand, antidepressant users who were 85 + years of age (OR: 1.98; 95% CI: 1.14–3.45), and had two or more comorbidities (OR: 2.02; 95% CI: 1.16–3.55) were more likely to report a disability.

**TABLE 2:** The association of CRN on patients’ reported health outcomes.

	General health status			Disability status		
* *	OR	95% (CI)		OR	95% (CI)	
**CRN**						
No	(Ref)			(Ref)		
Yes	0.67	0.39	1.16	1.34	0.83	2.14
**Age group**						
65–74	(Ref)			(Ref)		
75–84	1.19	0.72	1.95	1.34	0.90	2.00
85+	1.62	0.83	3.17	1.98	1.14	3.45
**Gender**						
Female	(Ref)			(Ref)		
Male	0.50	0.30	0.83	1.21	0.79	1.84
**Race**						
Non-Hispanic White	(Ref)			(Ref)		
Non-Hispanic Black	0.46	0.22	0.97	1.65	0.77	3.54
Hispanic	1.05	0.34	3.31	1.66	0.52	5.35
Others	5.77	0.64	52.12	2.21	0.43	11.40
**Education**						
Less than high school	0.86	0.49	1.50	1.15	0.69	1.93
High school graduate	(Ref)			(Ref)		
Some college degree	2.48	1.23	5.02	0.60	0.35	1.02
College graduate	3.01	1.57	5.78	0.61	0.38	0.97
**Marital status**						
Single	0.95	0.50	1.81	0.98	0.58	1.66
Married	(Ref)	—	—	(Ref)		
Widowed	0.91	0.51	1.63	1.46	0.92	2.32
**Income (US$)**						
<10,000 per year	(Ref)	—	—	(Ref)		
10,001–20,000 per year	1.09	0.52	2.28	0.93	0.44	1.96
20,001–40,000 per year	1.90	0.85	4.25	0.50	0.23	1.06
≥40,001 per year	3.01	1.22	7.42	0.40	0.18	0.89
**Census region**						
Northeast	(Ref)			(Ref)		
Midwest	1.21	0.63	2.32	0.96	0.55	1.67
South	2.01	1.08	3.75	0.76	0.46	1.27
West	1.21	0.59	2.50	0.94	0.52	1.70
**Residence**						
Non-metropolitan	(Ref)			(Ref)		
Metropolitan	0.81	0.49	1.33	1.07	0.71	1.63
**CCI**						
0	(Ref)			(Ref)		
1	0.34	0.13	0.87	1.31	0.64	2.67
2+	0.32	0.14	0.72	2.02	1.16	3.55
Unknown	0.61	0.27	1.33	1.38	0.79	2.39

CRN, cost-related medication nonadherence; CCI, charlson comorbidity index.

After controlling for other covariates, CRN was statistically associated with outpatient visits (OR: 1.89; 95% CI: 1.19–3.02) but not statistically associated with ER visits (OR: 1.10; 95% CI: 0.69–1.76) (Table 3). The factors associated with outpatient visits, antidepressant users with an income of $10,001-$20,000 per year (OR: 2.28; 95% CI: 1.07–4.84), an income of $20,001-$40,000 per year (OR: 2.62; 95% CI: 1.20–5.74), an income of more than $40,000 per year (OR: 2.66; 95% CI: 1.15–6.15), had two or more comorbidities (OR: 2.06; 95% CI: 1.18–3.60) were more likely to visit the outpatient department. On the other hand, antidepressant users who were age 85+ (OR: 0.56; 95% CI: 0.32–0.99), were living in the South region (OR: 0.54; 95% CI: 0.32–0.89), were metropolitan residence (OR: 0.41; 95% CI: 0.27–0.63) and had one comorbidity (OR: 0.99; 95% CI: 0.48–2.04) were less likely to visit outpatient department. Moreover, the factors associated with ER visits, antidepressant users were age 85+ (OR: 1.99; 95% CI: 1.16–3.42) were more likely to visit ER. By contrast, antidepressant users who had an income of $20,001-$40,000 per year (OR: 0.30; 95% CI: 0.14–0.62), or an income of more than $40,000 per year (OR: 0.37; 95% CI: 0.17–0.80) were less likely to visit ER.

**TABLE 3:** The association of CRN on patients’ healthcare Utilization.

	Outpatient visit			ER visit		
	OR	95% (CI)		OR	95% (CI)	
**CRN**						
No	(Ref)			(Ref)		
Yes	1.89	1.19	3.02	1.10	0.69	1.76
**Age Group**						
65–74	(Ref)			(Ref)		
75–84	0.92	0.62	1.36	1.36	0.91	2.03
85+	0.58	0.33	1.02	1.99	1.16	3.42
**Gender**						
Female	(Ref)			(Ref)		
Male	0.71	0.47	1.07	1.49	0.99	2.24
**Race**						
Non-Hispanic White	(Ref)			(Ref)		
Non-Hispanic Black	1.63	0.79	3.36	1.72	0.85	3.51
Hispanic	0.85	0.26	2.73	0.51	0.18	1.48
Others	1.31	0.31	5.47	0.48	0.11	2.12
**Education**						
Less than high school	1.32	0.79	2.21	0.71	0.43	1.17
High school graduate	(Ref)			(Ref)		
Some college degree	1.17	0.69	1.98	0.67	0.39	1.14
College graduate	1.07	0.66	1.72	0.73	0.45	1.17
**Marital Status**						
Single	0.86	0.51	1.45	0.87	0.51	1.46
Married	(Ref)			(Ref)		
Widowed	0.84	0.53	1.34	0.79	0.50	1.26
**Income (US$)**						
<10,000 per year	(Ref)			(Ref)		
10,001–20,000 per year	2.28	1.07	4.84	0.59	0.30	1.18
20,001–40,000 per year	2.62	1.20	5.74	0.30	0.14	0.62
≥40,001 per year	2.66	1.15	6.15	0.37	0.17	0.80
**Census Region**						
Northeast	(Ref)			(Ref)		
Midwest	0.97	0.57	1.67	0.89	0.52	1.53
South	0.54	0.32	0.89	0.68	0.41	1.12
West	0.82	0.46	1.46	0.92	0.52	1.64
**Residence**						
Non-metropolitan	(Ref)			(Ref)		
Metropolitan	0.41	0.27	0.63	1.04	0.69	1.58
**CCI**						
0	(Ref)			(Ref)		
1	0.99	0.48	2.04	0.65	0.31	1.36
2+	2.06	1.18	3.60	1.65	0.96	2.85
Unknown	1.95	1.13	3.37	1.20	0.70	2.06

CRN, Cost-related medication nonadherence; CCI, charlson comorbidity index; ER, emergency room.

## 4 Discussion

This study found that the prevalence of CRN was high among antidepressant users. The prevalence of CRN in antidepressant users is not well documented in the literature. Many studies reported the prevalence of CRN among individuals with depression, and it was relatively over 20% (Bambauer et al., 2007; Zivin et al., 2009; Gu and Shen, 2020). From the literature, gender was not typically associated with CRN. However, our study and other studies reported that females were more likely to experience CRN than males (Heisler et al., 2005; Briesacher et al., 2007; Zivin et al., 2010).

We found that CRN was not associated with general health status and disability status for self-reported health outcomes. In comparison to other studies, De Avila et al. reported a higher risk of persistent CRN was linked to worse self-reported health and depression (De Avila et al., 2021). Bambauer et al. indicated that CRN was worsened by poor health among both older adults and beneficiaries with disabilities (Bambauer et al., 2007). Furthermore, for self-reported healthcare utilization, CRN was associated with increased outpatient visits. The reason for increased outpatient visits may be related to the deterioration of the patient’s clinical outcome. There was a clear relationship between non-adherence and worsening patient clinical outcomes among individuals with antidepressant drugs (Ho et al., 2016). Also, our study found there was no association for ER visits and CRN. In comparison to other studies, Blanchard et al. indicated a statistically significant link between severe CRN and ER visits (Blanchard et al., 2013). Individuals who reported CRN were more likely to visit ER at least once and had a greater overall mean number of visits than those who did not report CRN (Blanchard et al., 2013).

Many sociodemographic characteristics were factors and played a major role in the association of self-reported healthcare utilization and self-reported health outcomes. Gender, education, income, and comorbidities were all factors of self-reported health status. Age, education, and income were all factors of self-reported disability status. Region, residence, and comorbidities were all factors of outpatient visits. Age, income, and comorbidities were all factors of ER visits. Healthcare providers need to consider these factors to enhance patient health status and healthcare efficiency.

Healthcare providers play a major role in influencing patients’ medication adherence. Medication adherence improves patient outcomes, and better patient outcomes are associated with close physician-patient relationships (Johnson, 2019). According to the patients, their healthcare provider is the most credible source of information about their current health condition and prescription regimen (Brundisini et al., 2015). Healthcare providers must pay close recognition to Medicare beneficiaries using antidepressants, in order to detect possible financial obstacles to adherence. Also, to support individuals in seeking other treatment options of antidepressants by identifying the causes of CRN and providing solutions to alleviate the financial burden on those impacted individuals. Solutions include offering less costly generic comparable drugs, drug-discount programs, and pharmaceutical and savings assistance programs (Bussell et al., 2017). Furthermore, interventions like medication therapy management (MTM) are necessary to avoid CRN in older adults since MTM assure that patients receive the best possible treatment. Patients with numerous chronic illnesses, complex drug regimens, high prescription prices, and multiple prescribers benefit the most from MTM (Community pharmacists and medication, 2021).

This study is the first of its kind to assess the association of CRN on self-reported healthcare utilization (ER visits and outpatient visits) and self-reported health outcomes (health status and disability status) among Medicare beneficiaries on antidepressants. In addition, this study uses the MCBS data, which has rich information about patients compared to using claims data only. The claims data has limited variables and is not rich in information. The MCBS data provides rich information on health status, access to services, insurance benefits, out-of-pocket costs, financial resources, socioeconomic information, and demographic information of Medicare beneficiaries.

There are three limitations of this study. First, this research did not distinguish between the various classes of antidepressants. The analysis did not include a subgroup analysis of antidepressant classes due to the small sample size of antidepressant users. Second, the causal relationship cannot be determined due to the nature of the cross-sectional study design. Exposure and outcome are both determined at the same time in cross-sectional studies (Kramer, 1988). We cannot assume that exposure happened before the outcome since exposure is assessed concurrently with the outcome (Kramer, 1988). Future studies using a longitudinal research design are required to establish cause-effect relationships. Finally, since MCBS was a self-reported survey, some of the assessments may be vulnerable to social desirability bias and recall bias – especially for CRN. However, MCBS surveyed Medicare beneficiaries three times a year to minimize recall bias (The Medicare Current Beneficiary Survey, 2021). Also, MCBS uses proxy measures if the respondent is unable to answer. Therefore, the use of self-reports is unlikely to result in significant recall bias.

## 5 Conclusion

For Medicare beneficiaries on antidepressants, CRN prevalence was high. Also, CRN contributed to more outpatient visits. The healthcare provider needs to define the reasoning for CRN and provide solutions to reduce the financial burden on the affected patient. Also, health care providers need to consider the factors that may enhance patient health status and healthcare efficiency.

## Data Availability

The data analyzed in this study is subject to the following licenses/restrictions: The data will not be made readily available for the privacy of the participants. Requests to access these datasets should be directed to https://www.cms.gov/Research-Statistics-Data-and-Systems/Research/MCBS.
