# Supplementary material for: Cost-Related Medication Nonadherence (CRN) on Healthcare Utilization and Patient-Reported Outcomes: Considerations in Managing Medicare Beneficiaries on Antidepressants
Source: Front Pharmacol. 2021 Dec 7;12:764697. doi: 10.3389/fphar.2021.764697 (PMC8688804; doi:10.3389/fphar.2021.764697)
Supplement: Supplementary file 1 [file Table1.DOCX]

**Appendix**

| **Antidepressant drugs** | |
| --- | --- |
| **Class** | **Generic Names** |
| **SSRIs** | Citalopram, Escitalopram, Fluoxetine, Fluvoxamine, Paroxetine, and Sertraline (6). |
| **SNRIs** | Desvenlafaxine, Duloxetine, Venlafaxine, Milnacipran, and Levomilnacipran (6). |
| **TCAS** | Amitriptyline, Desipramine, Doxepine, Nortriptyline, Imipramine, Amoxapine, Clomipramine, Trimipramine, Maprotiline, and Protriptyline (6). |
| **MAOIs** | Phenelzine, Selegiline, and Tranylcypromine (6). |
| **Atypical Antidepressants** | Bupropion, Mirtazapine, Nefazodone, Trazodone, Vilazodone, and Vortioxetine (6). |
